# Comments to Drs Taillefer de Laportalière, Jullien, Yrondi, Cestac, and Montastruc

**DOI:** 10.1017/S0033291723002490

**Published:** 2023-12

**Authors:** Dong-Jing Fu, Cynthia A. Bossie, Rosanne Lane, Adam Janik, Carla M. Canuso, Wayne C. Drevets

**Affiliations:** 1 Department of Neuroscience Clinical Development, Janssen Research & Development, LLC, Titusville, NJ, USA; 2 Department of Clinical Statistics, Janssen Research & Development, LLC, Titusville, NJ, USA; 3 Department of Neuroscience Clinical Development, Janssen Research & Development, LLC, San Diego, CA, USA

We read with interest the systematic review by Taillefer de Laportalière, Jullien, Yrondi, Cestac, and Montastruc (2023) in *Psychological Medicine* on the reporting of adverse events (AEs) in registration trials of esketamine. Based on their review, Taillefer de Laportalière et al. (2023) suggested that the quality of AE reporting in 10 peer-reviewed manuscripts of esketamine clinical trials (published across six different journals between 2018 and 2021) was poor; they also remarked that AEs were reported less frequently in journal publications than in ClinicalTrial.gov Registers. We recognize the paramount importance of transparency and accuracy in the reporting of safety data, and thus welcome the opportunity to provide clarification herein to the authors' review.

From 2010 to present day, the host journals for the 10 published esketamine manuscripts required that authors complete and submit the main CONsolidated Standards Of Reporting Trials (CONSORT) checklist (including one item on reporting of harms) (Schulz, Altman, Moher, & CONSORT Group, 2010) along with the manuscript. The journals reviewed the checklist to assure compliance with the EQUATOR standards for transparent, high-quality reporting of clinical trial findings. In contrast, Taillefer de Laportalière et al. (2023) used a 21-item checklist, focused solely on AEs, that was adapted from the CONSORT Extension of Harms (Ioannidis et al., 2004), and based on their scoring (0 or 1 point per item; equal weighting of items) concluded that AE reporting was poor. Of note, their AE checklist was not required by any of the journals where the esketamine clinical trial data were published (i.e. *JAMA Psychiatry*, *American Journal of Psychiatry*, *Journal of Clinical Psychiatry*, *International Journal of Neuropsychopharmacology*, *American Journal of Geriatric Psychiatry*, *BMC Psychiatry*).

In the same month that Taillefer de Laportalière et al. (2023) published their review of AEs, the CONSORT Harms Group published an updated guideline for the reporting of harms in randomized trials (Junqueira et al., 2023). In this updated guideline, Junqueira et al. (2023) acknowledge that there are ‘major challenges in fully reporting diverse AEs within a limited amount of space in journal articles.’ The CONSORT Harms Group went on to ‘define harms as the totality of possible adverse consequences of an intervention or therapy.’ They state that ‘harms might be assessed systematically via measuring variables for all participants using standardized clinical examinations, questionnaires, and medical instruments,’ and non-systematically based ‘on the passive or unstructured reporting of AEs.’

Notably, our reporting of AEs and harms in the esketamine clinical trial manuscripts was generally consistent with these updated, enhanced recommendations insofar as authors of the esketamine manuscripts included not only a summary of reported AEs, but also the results from a full array of systematically evaluated quantitative safety assessments. For example, the TRANSFORM-2 publication (manuscript and Supplementary Material; Popova et al., 2019) includes Modified Observer's Assessment of Alertness/Sedation score as a measure of sedation, blood pressure changes, clinically significant ECG changes, suicidal ideation based on the Columbia Suicide Severity Rating Scale, dissociation based on the Clinician Administered Dissociative States Scale, withdrawal symptoms after cessation of esketamine assessed by the Physician Withdrawal Checklist, and outlier data for sedation, dissociation, and treatment-emergent blood pressure increase. Nevertheless, Taillefer de Laportalière et al. (2023) reported this manuscript to be of low quality for reporting of harms (in their Table 2). Taillefer de Laportalière et al. (2023) focused their analysis solely on reported AEs without mentioning data from these systematic safety assessments. Our inclusion of data from all of the abovementioned safety measures, however, yielded a broader clinical perspective of ‘harms,’ consistent with the updated recommendations by the CONSORT Harms Group (Junqueira et al., 2023).

Furthermore, in the Taillefer de Laportalière et al. review (2023), the comment that AEs were reported less frequently in journal publications than in the ClinicalTrial.gov Registers is not unexpected, given that reporting of AEs within a clinical manuscript and reporting of AEs in ClinicalTrials.gov have different objectives/scopes and are guided by different policies/instructions. Studies registered in ClinicalTrials.gov have a unique record with a structured template into which study information/data are uploaded. The information/data contained in ClinicalTrials.gov are not meant to be identical to those in journal publications for the same trial; instead, the two sources often contain partial overlap in presentation of safety outcomes.

By way of example, for most studies the AE data presented in published esketamine manuscripts focused on AEs and serious AEs reported in *the double-blind treatment periods* of the randomized, controlled trials, providing context for AEs with emphasis on the events of clinical importance. A ClinicalTrials.gov identifier was included in each of the 10 esketamine manuscripts, thereby providing interested readers with more detailed information on many aspects of each study, including AEs in all study phases that may not have been included in the published manuscripts. In contrast, in Clinical Trials.gov, esketamine AE data are presented for *all study periods*, without the option to provide clinical context, and depending on the study design may include the follow-up period during which participants did not receive esketamine (which is of limited clinical significance for drug-related AEs given esketamine's short elimination half-life). For example, in the relapse prevention study, Daly et al. (2019) reported treatment-emergent AEs during the double-blind *maintenance* phase, which aligned with the primary objective and primary efficacy endpoint of the study and was the most informative study phase because it included a control arm and was performed under double-blind conditions. In contrast, per the template, the ClinicalTrials.gov Register for the study not only reports AEs for the maintenance phase, but also for the open-label induction phase as well as the optimization and follow-up phases.

Finally, we note several inaccuracies in the review by Taillefer de Laportalière et al. (2023), some of which we list here. Firstly, the authors state that esketamine's approval by the United States Food and Drug Administration (FDA) and the European Medicines Agency (EMA) was based on four trials of which only one reached statistical significance. This information is incorrect; statistically significant superiority on the pre-specified primary efficacy endpoint was achieved in two of the studies considered pivotal (TRANSFORM-2 and SUSTAIN-1), supporting esketamine's approval for treatment-resistant depression by the FDA and EMA and by regulatory authorities in >70 countries worldwide (Daly et al., 2019; Popova et al., 2019; Spravato Prescribing Information, 2020; Spravato Summary of Product Characteristics, 2023). Secondly, the Clinical Trials.gov data cited in Table 3 within the column labeled ‘patients with at least 1 AE’ are incorrect (Taillefer de Laportalière et al., 2023). In Clinical Trials.gov, the corresponding data are presented as the total number of patients affected by any non-serious AE above a frequency threshold. Thirdly, the data for number of patients who discontinued treatment due to an AE in Table 3 of the review article are incomplete (but are correctly provided by treatment group and study phase in the CONSORT-compliant patient disposition figure for each of the 10 manuscripts).

In concluding remarks, Taillefer de Laportalière et al. (2023) suggested that post-marketing studies of esketamine are needed. Janssen already has been committed to continuous assessment of esketamine's safety profile through *post hoc* analysis of safety data from the clinical development studies (Chen et al., 2022; Citrome, DiBernardo, & Singh, 2020; Doherty et al., 2020; Doty et al., 2021; Katz et al., 2021; Williamson et al., 2022, 2023) as well as comprehensive post-marketing pharmacovigilance surveillance from multiple sources. Moreover, Janssen follows an FDA-mandated ongoing Risk Evaluation and Mitigation Strategy (REMS) (Spravato REMS, 2023) to mitigate potential risks of serious adverse outcomes resulting from sedation, dissociation, and misuse/abuse of esketamine, thereby ensuring that the benefits of esketamine outweigh its risks. Real-world safety findings from the REMS have been presented at medical conferences, beginning in 2020 (Starr et al., 2020), and most recently in 2023. Also, Janssen's long-term safety trial, SUSTAIN-3, was recently completed; a manuscript reporting interim results, which includes up to 4.5 years (2769 cumulative patient-years) of continued esketamine treatment, was recently published (Zaki et al., 2023). Final results are being presented at 2023 medical conferences, published, and posted on Clinical Trials.gov to inform on long-term safety.

Based on their focused evaluation of AEs (without regard to clinical relevance), but lacking other systematically measured safety outcomes reported in the peer-reviewed esketamine literature, Taillefer de Laportalière et al. (2023) suggested that ‘the benefits/risks balance of esketamine … is flawed due to the poor accuracy and completeness of harm data.’ We strongly disagree with this statement and stand by the transparency and accuracy of our reporting of study data, in alignment with the CONSORT recommendations. To the contrary, all data (in esketamine publications and in Clinical Trials.gov) that Taillefer de Laportalière et al. (2023) included in their review are reported/provided by Janssen, with the aim of full disclosure of accurate safety data. Furthermore, readers can submit a request for study data via a URL link that is provided within the published manuscripts.
